# Biases in Ecoacoustics Analysis: A Protocol to Equalize Audio Recorders

**DOI:** 10.3390/s24144642

**Published:** 2024-07-17

**Authors:** Andrea Potenza, Valentina Zaffaroni-Caorsi, Roberto Benocci, Giorgia Guagliumi, Jalal M. Fouani, Alessandro Bisceglie, Giovanni Zambon

**Affiliations:** 1 Department of Earth and Environmental Sciences, University of Milano-Bicocca, 20126 Milan, Italy; valentina.zaffaronicaorsi@unimib.it (V.Z.-C.); roberto.benocci@unimib.it (R.B.); g.guagliumi@campus.unimib.it (G.G.); alessandro.bisceglie@unimib.it (A.B.); giovanni.zambon@unimib.it (G.Z.); 2 Independent Researcher, 40132 Bologna, Italy

**Keywords:** soundscape, equalization, eco-acoustic indices, recorder, biases

## Abstract

Eco-acoustic indices allow us to rapidly evaluate habitats and ecosystems and derive information about anthropophonic impacts. However, it is proven that indices’ values and trends are not comparable between studies. These incongruences may be caused by the availability on the market of recorders with different characteristics and costs. Thus, there is a need to reduce these biases and incongruences to ensure an accurate analysis and comparison between soundscape ecology studies and habitat assessments. In this study, we propose and validate an audio recording equalization protocol to reduce eco-acoustic indices’ biases, by testing three soundscape recorder models: Song Meter Micro, Soundscape Explorer Terrestrial and Audiomoth. The equalization process aligns the signal amplitude and frequency response of the soundscape recorders to those of a type 1 level meter. The adjustment was made in MATLAB R2023a using a filter curve generated comparing a reference signal (white noise); the measurements were performed in an anechoic chamber using 11 audio sensors and a type 1 sound level meter (able to produce a .WAV file). The statistical validation of the procedure was performed on recordings obtained in an urban and Regional Park (Italy) assessing a significant reduction in indices’ biases on the Song Meter Micro and Audiomoth.

## 1. Introduction

The production of pocket digital recorders in the past few decades allowed researchers and amateurs to record many hours of environmental noise and sounds [1]. These recorders along with mobile phones have been implemented by researchers into their work and nowadays are used in various acoustic research branches (e.g., noise mapping [2,3], soundscape assessments [4,5,6,7], bio-acoustics research and sound event detection through machine learning [8,9]).

This study focuses on an equalization procedure to reduce biases in the comparison of audio recorders which can be used in all the disciplines mentioned before. However, in this study, the evaluation of the procedure will be carried out focusing on soundscape ecology given the authors’ main field of research (soundscape ecology). Therefore, the term “soundscape” will be used in the terms of this discipline from here on.

Soundscape ecology studies witnessed remarkable growth in the past few decades [10] thanks to new technologies that allowed for the production of different autonomous passive recorders [11]. These developments boosted the investigation of soundscape environments (terrestrial and maritime) and the role of anthropogenic noise on habitat degradation and biodiversity loss.

The exploration of soundscapes involves the application of passive acoustic monitoring (PAM), a method that entails deploying recording devices in the studied area and retrieving them after days or weeks. PAM serves various purposes, from monitoring a particular taxon to recording sounds in a specific environment [11]. To achieve this, researchers employ terrestrial recorders with specific characteristics like sensitivity, frequency response, dynamic range and bandwidth limits. The analysis of recordings relies mainly on eco-acoustic indices [12,13,14] which allow for summarizing the audio information and classifying soundscapes by analyzing their pitch, saturation and amplitude. This process involves confronting time steps or frequency bins to discern patterns and variations [14]. Soundscape monitoring can be carried out in different spatial designs using a regular grid of devices or arrays of sensors operating in tandem [11,15]. While the majority of studies use one to three recorders, a minority deploy more than ten instruments in the field [15,16,17,18]. Given these monitoring spatial designs, the use of all available devices, regardless of the manufacturers, is an attractive strategy to expand the study area at reduced costs.

The main challenge of this wide array of available recording instruments lies in the potential introduction of biases since recorders of different brands will mostly produce audio files with diverse properties when exposed to the same soundscape (i.e., amplitude values over the spectrum). Moreover, these biases can also be present in devices of the same brand and model due to the inherent uncertainty in sensitivity and frequency response. For these reasons, comparing sound pressure levels (SPLs) or eco-acoustic indices’ values between recordings made with instruments located in different parts of a forest or different habitats can be problematic. Even when using a single-sensor model, these biases persist due to manufacturing variability, as assessed in [16] and declared by manufacturers in their user guides (i.e., sensitivity uncertainty range). The bias increases when different models are employed due to frequency response differences, wider sensitivity ranges, dynamic range and bandwidth limits. Furthermore, these biases may cause incongruities in indices’ values and trends if the comparison is performed between different areas using different recorders [19,20,21]. In fact, previous studies have evidenced that differences in the frequency response led to conflicting conclusions when using indices as proxies for biodiversity or soundscape characteristics [22]. Furthermore, it is common to observe a non-correspondence between eco-acoustic indices’ trends, like ACI values [19] in studies that compare eco-acoustic indices across different regions or of different habitats. In [21], Sethi and colleagues analyzed four diverse datasets (temperate forest in the USA, rainforest in Malaysia, agricultural tea landscape in Taiwan and grassland in India) investigating the correlation between acoustic features (i.e., convolutional neural network and 60 soundscape indices) and avian species richness. The calculated indices also included the acoustic complexity index (ACI), the acoustic diversity index (ADI), the bio-acoustic index (BI), the normalized difference soundscape index (NDSI) and the temporal and spectral entropy (Ht, Hf) [23]. As they reported, there was not a single eco-acoustic index correlated to avian richness across all datasets. The authors hypothesized that these incongruities may be due to comparing diverse habitats and ecosystems at different latitudes as well as using audio recorders of different brands or in different settings (i.e., spatial deployment) and conditions (i.e., rain protection bags).

To overcome these biases, it is crucial to equalize the audio files acquired from various recorders. Equalization involves processing these audio files using a curve derived from comparing two recordings of white noise: one obtained with the soundscape sensor and another with a type 1 sound level meter, which serves as reference. This process enables the generation of soundscape recordings with a “flat” frequency response (akin to that of the sound level meter), ensuring consistent pressure levels and eco-acoustic indices across soundscape devices.

The necessity of granting an accurate comparison between recorders has been previously recognized in other papers which proposed calibration methods or pipelines to study the spectral portions in which the recordings are comparable [24,25,26].

In [24], three procedures with various accuracy to calibrate audio files were proposed. Among these, the most effective approach involves calibrating the recording using a known frequency level as a reference value. Unfortunately, the different sensitivity curves of the instruments (which are not flat) do not allow for a perfect calibration, and thus, an equalization procedure is necessary.

In [25], the authors perform an equalization on one-third octave bands between 0.025 and 6.3 kHz. Their work is an initial step in facilitating accurate comparisons of soundscape recordings.

Finally, in [26], the authors proposed a pipeline to compare the eco-acoustic indices between Audiomoth and SM4 devices. In their article, Luna-Naranjo and colleagues addressed the inherent variability in these two devices mainly by selecting comparable frequency ranges between the two instruments (on which the eco-acoustic indices are calculated) and normalizing the signal amplitude.

In this study, we propose an equalization protocol aiming at mitigating biases inherent in soundscape recordings obtained from three different devices (Song Meter Micro, Audiomoth and Soundscape Explorer Terrestrial), by aligning these audio files to those captured by class 1 level meter (LD831-C), a standard reference device. This process entails adjusting the signal amplitude and frequency response of the soundscape recorders to match those of the level meter, ensuring consistency across the entire spectrum. Alongside the protocol proposal, its validation is carried out in this paper to show its efficacy. As a result, we aim to help researchers with further comparisons within and outside the study area with greater reliability and precision, enabling a more complete understanding of acoustic ecosystems and their dynamics.

This paper is structured around four key pillars, outlined as follows:

- An explanation of the equalization procedure’s steps;
- Recording white noise to calculate the equalization curves;
- The application of the equalization curves to the soundscape recordings;
- The validation of the procedure using three case studies.

## 2. Materials and Methods

In this section, we describe the instrumentation and steps followed for the development of the equalization process:

1. Obtaining white noise recordings to generate the equalization curves;
2. Defining the equalization procedure;
3. Validating the equalization process
  - On white noise measures (obtained in the lab);
  - In an in-field experiment placing the devices in a single measurement point;
4. Providing an example of a monitoring campaign carried out in a Regional Park using two different brands of devices.

### 2.1. Materials

**Figure 1:**
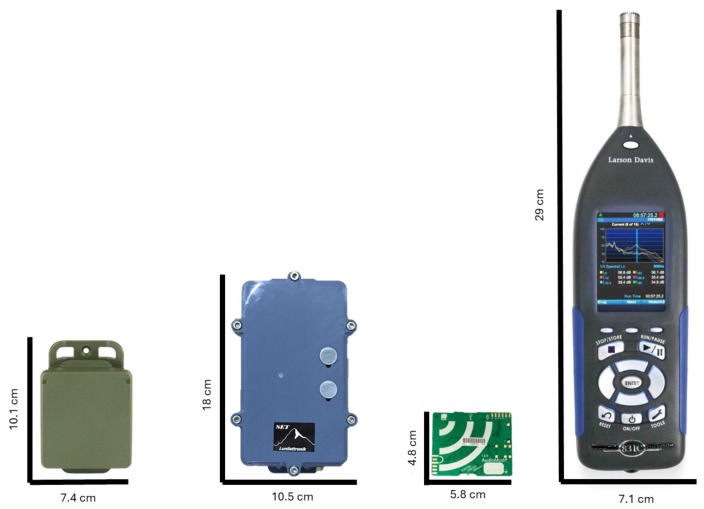
Devices employed in this study and relative dimensions (from left to right: Song Meter Micro, Soundscape Explorer Terrestrial, Audiomoth and LD831-C).

For the creation of this protocol, we considered three models of different brands of soundscape recorders and a sound level meter (Figure 1):

- Song Meter Micro (Wildlife Acoustics, Inc., Maynard, MA, USA);
- Soundscape Explorer Terrestrial (Lunilettronik Coop S.p.a., Fivizzano, Italy);
- Audiomoth (Open Acoustic Devices, Oxford, UK);
- LD831-C (Larson-Davis, Depew, NY, USA).

These models were chosen given their diffusion in the soundscape ecology community, their relative economical cost and the different sensitivity curves. We used a class 1 level meter to obtain the best input result.

**Figure 2:**
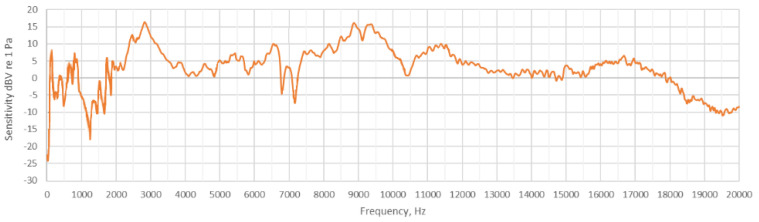
Sensitivity curve for Song Meter Micro. The sensitivity curve is referred to the whole signal transmission chain (provided by Wildlife Acoustic Support Team).

The Song Meter Micro (SMM hereafter) is a passive recorder with a maximum sampling rate of 96 kHz. The sensitivity of the whole signal transmission chain (i.e., microphone, gain and analog-to-digital converter) is 2 dBV ± 4 dBV relative to 1 Pa at 1 kHz Full-Scale, measured using a gain of +18 dB. The sensitivity curve is not linear with frequency (Figure 2). It generates output files in wave format, 16-bit and not compressed. The maximum recording length is 60 min. It works using three AA batteries. The model tested here is the first version of the Song Meter Micro series.

**Figure 3:**
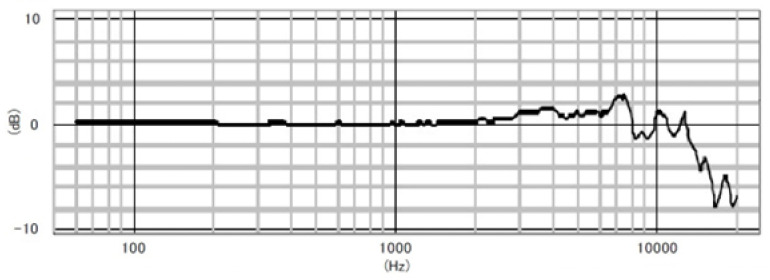
Microphone sensitivity curve for Soundscape Explorer Terrestrial (Adapted from the Microphone technical data by PRIMO Co., Ltd., Tokyo, Japan, installed on the SET).

The Soundscape Explorer Terrestrial (SET hereafter) is a programmable recorder equipped with two microphones (with a sampling rate of 48 and 192 kHz, respectively) and environmental sensors (for humidity, temperature, light and atmospheric pressure). The microphone has a sensitivity of −28 dBV ± 3 dBV relative to 1 Pa at 1 kHz; its frequency response is almost flat up to 6 kHz (Figure 3). It generates output files in wave format, 16-bit and not compressed. It works using eight AA batteries.

**Figure 4:**
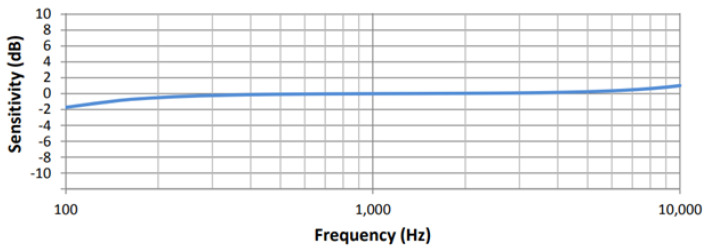
Microphone sensitivity curve for Audiomoth 1.2.0 (Adapted from RevSpace).

The Audiomoth 1.2.0 (AM hereafter) is a programmable, low-cost recorder equipped with an analog MEMS (Micro Electrical–Mechanical System) microphone with a maximum sampling rate of 384 kHz. The sensitivity of the microphone is −38 dBV ± 6 dBV relative to 1 Pa at 1 kHz, and its frequency response is almost flat (without the waterproof case) (Figure 4). An analysis of the response with and without the waterproof case is carried out. Its output files are .wav, 16-bit and not compressed. It runs using three AA batteries.

Finally, we used as a reference device the sound level meter of class “A” LD-831C (831 hereafter) produced by Larson-Davis. It is equipped with a class A microphone, calibrated in a certified laboratory, with a sensitivity of −26.19 dBV relative to 1 Pa at 251.2 Hz. Moreover, it has a flat response over all frequencies in the interval 0–20 kHz. This level meter can produce an uncompressed .wav file, 16-bit.

### 2.2. Methods

#### 2.2.1. Recording White Noise in the Anechoic Chamber

The first step of the equalization process involves acquiring white noise recordings using the soundscape devices and the level meter simultaneously.

These measurements were carried out in an anechoic chamber, 4 × 4 m and 2.8 m high, configured to operate in “full anechoic” mode. The chamber can also operate in the “semi-anechoic” configuration (walkable floor without pyramid absorbers), but the “full anechoic” mode ensures an optimal background noise level of 10 dBA for precise recordings. Finally, the cut-off frequencies are around 120 Hz due to the size of the chamber and of the sound-absorbing elements.

**Figure 5:**
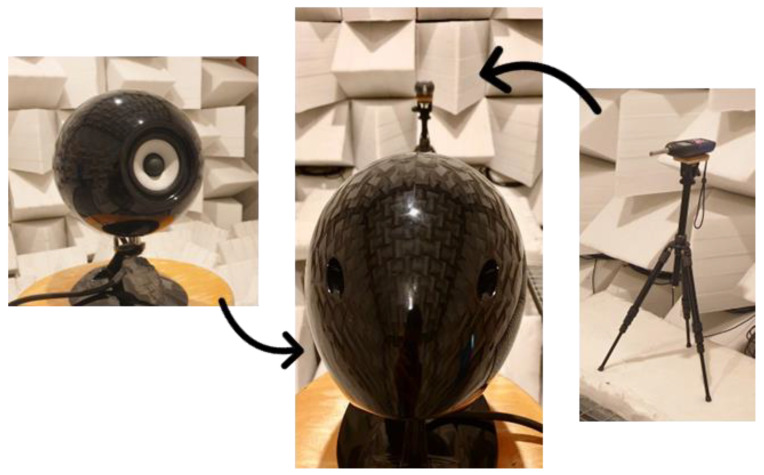
White noise measurements in the anechoic chamber with a loudspeaker and LD831-C.

The reference signal (white noise) was built using Audacity 3.0.2 [27] with a sample rate of 48 kHz, RMS value of −18.74 dBFS and a duration of 20 s. To reproduce the signal in the anechoic chamber, we employed the loudspeaker TD508 Mk3 of the Eclipse series (Denso Ten, Kobe, Japan) (Figure 5). This speaker has a sensitivity of 82 dB/W/m, emitting between 52 Hz and 27 kHz with an angular coverage of +15/−10°.

Measurements were performed by placing one device at a time in the same position: 1 m from the loudspeaker, with the microphone facing the sound source and vertically centered in the middle of the woofer (Figure 5).

**Figure 6:**
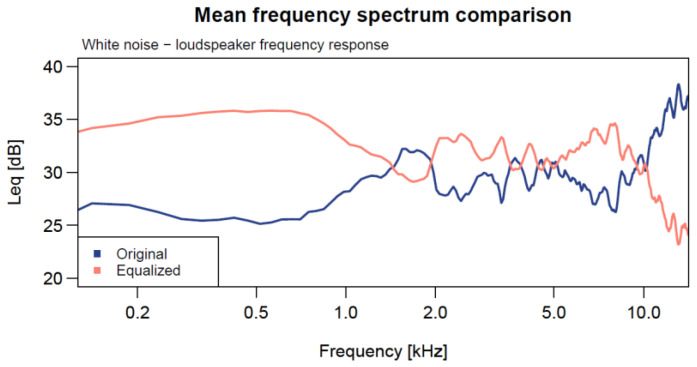
White noise mean frequency spectrum showing the original frequency response of the loudspeaker (blue) and equalized (orange) to obtain a white noise signal measured by LD831-C.

The appropriate functioning and frequency response of the loudspeaker were tested previous to the measurements. The results showed that the white noise emitted by the device was not flat (Figure 6). For this reason, the loudspeaker output was recorded with the level meter and equalized to generate a new output in the range 24–15 kHz that was perfectly flat when measured with the level meter. The equalization of the loudspeaker was performed using the white noise generated with Audacity and the signal recorded with the level meter when emitted by the loudspeaker.

The following settings were used to measure the white noise with the soundscape recorders (Table 1). To understand intra-model variability, we tested all the devices available in our laboratory and some kindly lent to us by Professor Guarnaccia of the Università degli Studi di Salerno; the devices were set as usually employed in the field by the research groups. All devices were set with parameter values used by our group during in-field monitoring [13,16,28,29]. The sampling frequency was set at 48 kHz to correctly sample all birds’ vocalizations. The amplitude gains were set for SMM at +18 dB since this is the default value suggested by Wildlife Acoustic, for SET, two values were analyzed due to in-field choices performed by the group during the years and the AM was set at “medium” (+15 dB) since it is the nearest value to the other device’s amplitude gains. The level meter gain was set to +0 dB since the device choice is between 0 or +20 dB. Finally, the level meter was employed without the windproof cap since the measurement was performed in a closed environment, while the AM was analyzed with and without the waterproof case to better understand its effect on the sensitivity curve.

#### 2.2.2. Equalization Procedure

The equalization procedure consists of calculating an equalization curve using the white noise measures and then applying it to in-field recordings. The process is carried out in the MATLAB environment.

##### Calculating the Equalization Curve

**Figure 7:**
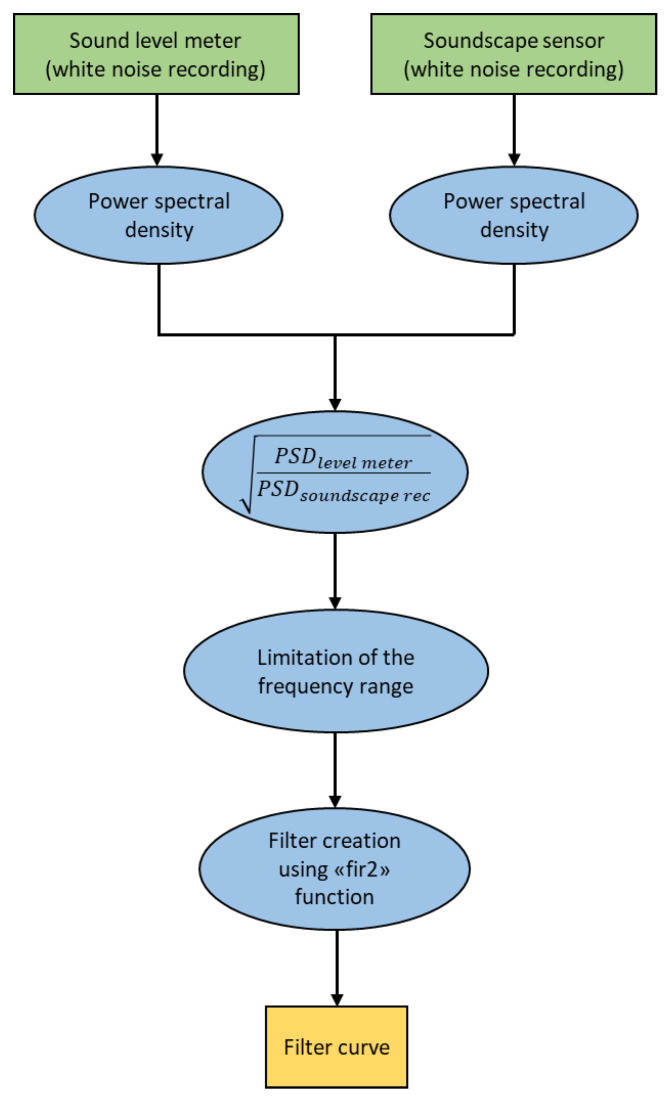
A scheme illustrating the steps to calculate the equalization curves.

The process to generate the equalization curves is summarized in Figure 7, and the MATLAB script is available in the Supplementary Materials Section. This procedure is an amended version of the one conducted in [30] for the aim of this study and to be able to run it through MATLAB R2023a [31].

The curve is used to generate a filter in the frequency domain which is applied to the in-field recordings.

The first step consists of calculating the power spectral density (PSD) of the level meter and soundscape white noise recordings, which describes the frequency distribution of the signal’s power [32].

These vectors are compared by dividing the PSD of the level meter by the soundscape device one.

The curve is finally calculated using the “fir2” function. This function returns an nth-order filter with frequency–magnitude characteristics by interpolating the desired frequency response onto a dense grid and then using the inverse Fourier transform and a Hamming window. The input parameters are the ratio between the PSDs, the number of FFT points and the frequency vector.

The equalization curve is calculated for each device since the frequency response is diverse even between recorders of the same brand.

Furthermore, considering that the soundscape devices have amplitude gain (Table 1), the white noise recorded by the 831 was amplified to +18 dB before computing the equalization curves. This gain was applied to avoid altering the gains set in the soundscape devices which are optimized to record distant sound events such as birds’ vocalizations. This operation was implemented using Audacity 3.0.2, and a rapid evaluation showed that the gain was linearly applied on all frequencies without any distortion on the recordings. To ensure an accurate comparison of the devices after the equalization, the calculation of the curves was performed on a single white noise 831 recording amplified at +18 dB.

##### Equalization of In-Field Recordings

**Figure 8:**
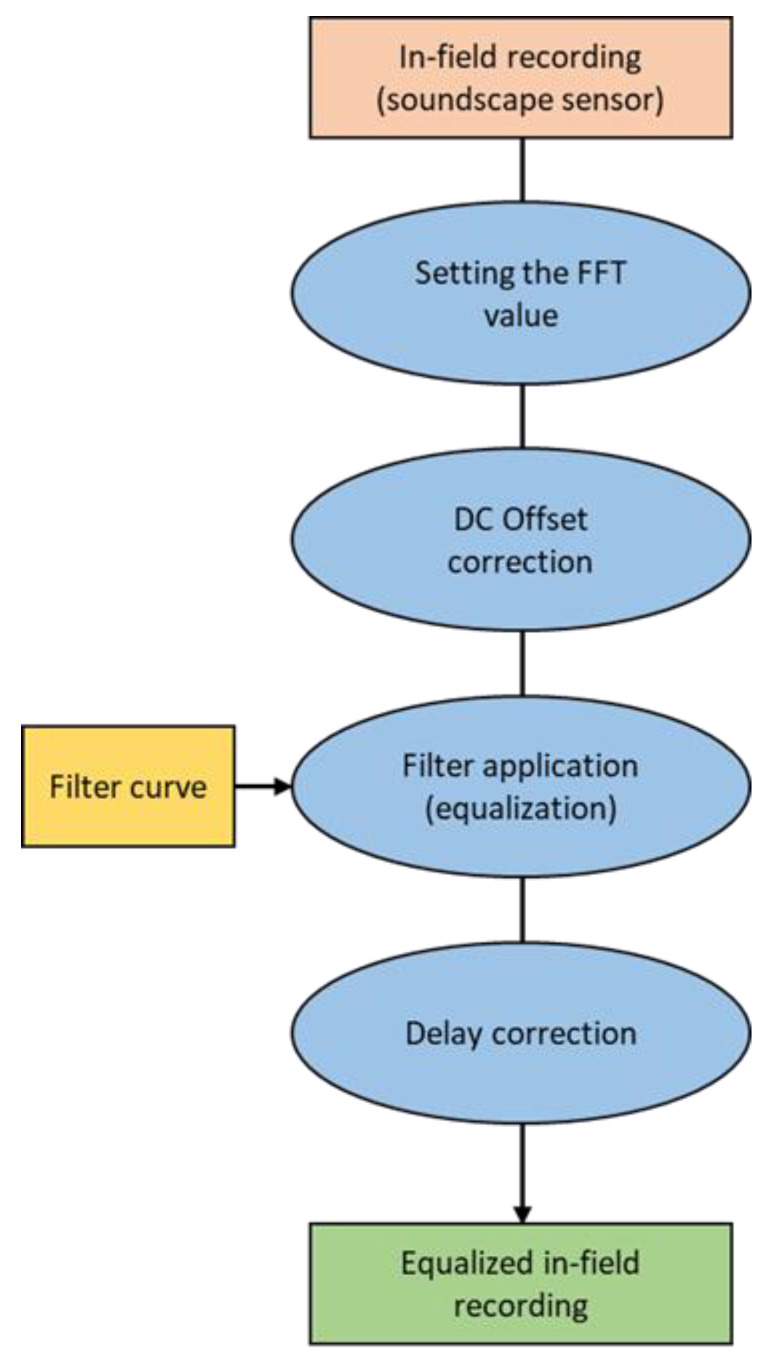
A scheme illustrating the equalization of an in-field recording.

After the implementation of the equalization, the curves were applied to the in-field recordings (using a MATLAB script available in the Supplementary Materials Section) following the scheme described in Figure 8.

The first step consists of the correction of the possible presence of the DC offset. This fixed voltage offset present in the audio chain of the device is visible as a shift of the waveform from the 0.0 horizontal center. It affects the calculation of the eco-acoustic indices since it alters the frequency domain (being a direct current, it manifests as an intensity peak in the first frequency bin of the signal). The removal of the DC offset is conducted by subtracting the offset to the signal; the offset value is determined utilizing the mean estimation method, wherein it corresponds to the mean value of the audio signal [33].

The second passage is the application of the equalization curve to the field recording. It is performed using a rational transfer function (“filter”) defined by the numerator and denominator coefficients; the numerator is set to 1, while the denominator is the result of the “fir2” function, in other words, the equalization curve.

Finally, since filtering a signal introduces a delay (i.e., the output signal is shifted in time), the delay is calculated using the “grpdelay” function and then corrected. This function calculates the delay of a narrow-band “group” of sinusoidal components; if the filter has a linear phase response, the group delay and phase delay are identical [34].

The equalized recording is finally saved in .wav format.

##### Parameters Set in the Equalization Script

**Figure 9:**
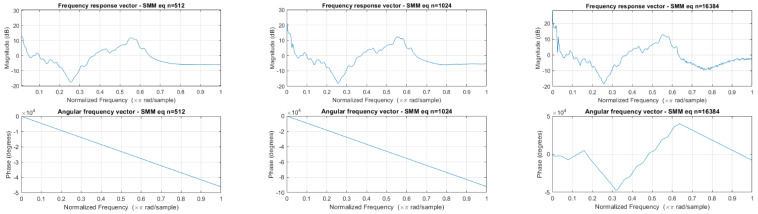
The frequency response vector and angular frequency vector of the equalization curves.

In the calculation of the equalization curve script, the number of FFT points was given in input to calculate the power spectral density. The number of FFT points was also applied to implement the curve using the “fir2” function; in particular, it was set as the filter’s order value. After analyzing their frequency response vector and angular frequency vector (Figure 9), we validated the equalization procedure using values of 512, 1024 and 16,384, since they allowed for good accuracy without excessive phase changes. This choice allowed us to obtain three calculated curves for each device.

The delay induced by the “filter” function consists of milliseconds of no-audio track placed at the beginning of the recording, shifting the start of the audio without increasing the time duration of the in-field file and thus losing the last recorded milliseconds. Given the linearity of the phase responses (Figure 9), the group delay and phase delay are identical [34]. The delay correction cuts those first milliseconds inserted by the filter, causing a reduction in the length of the recording; this reduction depends on the delay which depends on the order value: it ranges from 0.0053 s using a value of 512 to 0.17 s using a value of 16,384.

#### 2.2.3. Validation of Equalization Process and Practical Example

**Figure 10:**
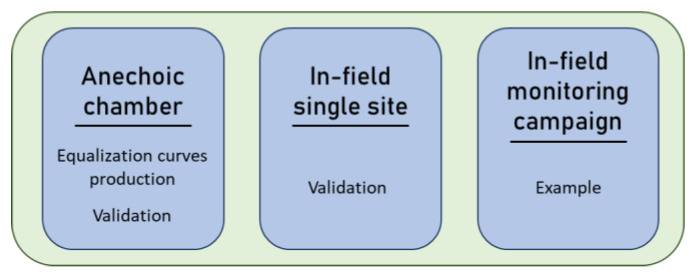
A scheme of the process explained in the following sub-sections.

The validation process (Figure 10) consists of evaluating the effects on the soundscape ecology analysis introduced by the proposed equalization procedure. Focusing on the three soundscape recorder models, it aims at identifying the best filter order to be used.

This validation process was carried out as follows:

- On white noise measures (anechoic chamber measurements);
- In an in-field experiment placing the devices at a single measurement point (urban park).

A practical example was used to better understand the effects of the procedure on eco-acoustic indices’ time trends derived from a monitoring campaign carried out in a Regional Park using two different brands of devices (SMM and SET also used in the previous phases of the validation process).

These three environments were considered due to their different characteristics:

- The anechoic chamber provides an ideal environment for recording identical signals across all devices, facilitating the calculation of equalization curves and enabling a precise comparison of a singular recording for each device.
- The monitoring in the urban park permits the evaluation of the effects of the equalization process in a real case scenario on nine 1 min recordings taken simultaneously at a single measurement site. This site is rich in traffic noise (i.e., cars, buses, motorcycles), birds’ vocalizations, cicadas’ vocalizations, human voices and airplane overflights; thus, the signals recorded are extremely diverse with events spanning the entire spectrum.
- The example of the Regional Park is proposed as a classic monitoring scheme example [15,16,35]. Its soundscape was assessed by placing nine devices on a regular grid (each point distant 200 m from the others), and the eco-acoustic indices were calculated by analyzing a 24 h time trend both before and after the equalization process. This real-world scenario serves as an excellent example for testing the efficacy of the proposed protocol.

##### Eco-Acoustic Indices

The analysis of recordings in soundscape ecology is carried out using, among other methods, the eco-acoustic indices [12,13,14]. The indices calculated to validate the equalization procedure are the following:

- ACI (acoustic complexity index): This quantifies the vocalizations of avifauna through the study of sound intensity modulation, which varies rapidly over time in the case of biophony but is very constant for numerous anthropogenic noises [14,36]. The implementation is based on the amplitude difference between adjacent time samples within a frequency band, relative to the total amplitude of that band [14].
- ADI (acoustic diversity index): This provides a measure of the diversity of intensity distribution in the spectrum by dividing it into time intervals and calculating the Shannon index [13,14]. Low values are due to extreme diversity in intensity distribution (i.e., nocturnal insects [14]) while high values to a distribution evenness (i.e., high levels of geophony and anthropophony [14] and bird species richness [13]).
- AEI (Acoustic Evenness Index): This is based on the same logic of ADI but applies the Gini coefficient instead of the Shannon index and consequently measures the inequality of signals in bands [37].
- BI (bio-acoustic index): This measures avian abundance by calculating the area under the mean frequency spectrum in the frequency range occupied by biophonies and characterized by a certain amplitude value (this threshold value, expressed in dB, is greater than the lowest value represented in the spectrum) [38].
- NDSI (normalized difference soundscape index): This assesses the distribution of the soundscape between anthropophony and biophony to estimate the level of anthropogenic disturbance of a habitat [39]. It is calculated by dividing the difference between biophony and anthropophony by their sum; the estimation of biophony and anthropophony is carried out by calculating the power spectral density in the frequency ranges of these soundscape components [39]. Values range in the [−1, +1] interval, where +1 ideally indicates the total dominance of biophonies and −1 the total dominance of anthropophonies [39,40].
- H (Acoustic Entropy): This provides an estimate of the total entropy, or heterogeneity, of the recording. It is calculated by computing the product of Shannon’s spectral entropy and temporal entropy [41]. Values range in [0, +1]; +1 indicates an even signal (i.e., silent recording or faint bird calls), while 0 indicates a pure tone (i.e., insects’ vocalizations) [14].
- DSC (Dynamic Spectral Centroid): This returns the spectral centroid of a recording (expressed in Hz) providing information about the sound events of a recording. It is calculated by dividing the spectrum in time intervals and computing the gravity center of the spectrum [13].
- ZCR (Zero-Crossing Rate): This measures the number of times per second that a signal crosses the instantaneous pressure of 0 and provides a measure of noisiness; high values are associated with noisy recordings and the presence of biophony, while low values are linked to tonal sounds [42,43].

These indices were calculated in the “R” environment (version 2023.03.0) [44] using the packages “seewave” and “soundecology”. For computing the DSC, a dedicated script was written [13]. The scripts of the ADI and AEI from the “soundecology” package were modified to add the minimum frequency values and thus mirroring the other eco-acoustic indices (ACI, BI; NDSI, DSC). The indices were calculated using an FFT value of 1024 which corresponds to a frequency resolution of FR = 46.875 Hz and a time resolution TR = 1/FR = 0.0213 s. The time duration was set as the minimum of the three FFT values (59.83 s).

For the white noise and pocket park recordings, the indices parameters were set as the following:

- ACI and DSC: min_freq = 500 Hz, max_freq = 12,000 Hz.
- ADI and AEI: min_freq = 500 Hz, max_freq = 12,000 Hz, freq_step = 10 Hz, dB_threshold = −50 dB.
- BI: min_freq = 1700 Hz, max_freq = 12,000 Hz.
- NDSI: min_anthro_freq = 500 Hz, max_anthro_freq = 1700 Hz, min_bio_freq = 1700 Hz, max_bio_freq = 12,000 Hz.
- H and ZCR: the entire spectrum.

The minimum frequency was set to 500 Hz due to the devices’ low sensitivity to low frequencies. In fact, even if the equalization process levels out the audio recordings to the one of a level meter, their original sensitivity to low frequencies does not allow for the optimal recording of sounds, and therefore, it is not possible to fully reproduce the fidelity of a sound level meter. For this reason, the minimum frequency was set at 500 Hz.

For the Regional Park monitoring, the indices’ parameters were the same except for the ADI and AEI for which the minimum frequency was set to 0 Hz and the dB_threshold to −50 dB.

##### Statistical Test

Regarding the simultaneous recordings acquired in the urban park, a statistical test was performed to assess the equivalence in the eco-acoustic indices derived from both the sound level meter and the soundscape devices (distinguishing between pre-processed and post-processed using 1 k, 16 k and 32 k FFT points). Given the simultaneity of the recordings, a pairwise test was chosen. Prior to conducting the pairwise Student’s test, Shapiro’s test and Bartlett’s test were employed to verify the assumptions of normality and homoscedasticity [45]. The indices that did not meet the assumptions were analyzed using the pairwise Wilcoxon test. The null hypothesis H0 of the Student and Wilcoxon test is that the mean difference is equal to 0; it is refused when *p*-value < 0.05.

##### White Noise (Anechoic Chamber)

The validation was first performed on the white noise recorded in the anechoic chamber. The recordings are not affected by the DC offset, probably due to the lack of humidity in the environment which does not alter the conductivity of the electronic circuit.

Two parameters were computed:

- The root-mean-square error (RMSE) of the amplitude (1)
  (1) $${\mathrm{RMSE}}_{\mathrm{amp}}=\sqrt{\frac{1}{n_{f}}\sum_{i=f_{min}}^{f_{max}}{\left(Amp_{slm}\left(f_{i}\right)-Amp_{recorder}\left(f_{i}\right)\right)}^{2}}$$

This equation compares the soundscape recording to the level meter one (which serves as reference). It was performed on the entire spectrum, subtracting the amplitude of the soundscape recorder (recorder) from the one of the level meter (“slm”). It was then normalized by the number of frequency bins and rooted.

- Percentage error on the eco-acoustic indices (ACI, ADI, AEI, BI, NDSI, H, DSC, ZCR) using the sound level meter as reference (2)
  (2) $$\%{\mathrm{error}}_{\mathrm{index}}=\frac{Index_{slm}-Index_{recorder}}{Index_{slm}}$$

##### Field Recordings at Bicocca Urban Park

**Figure 11:**
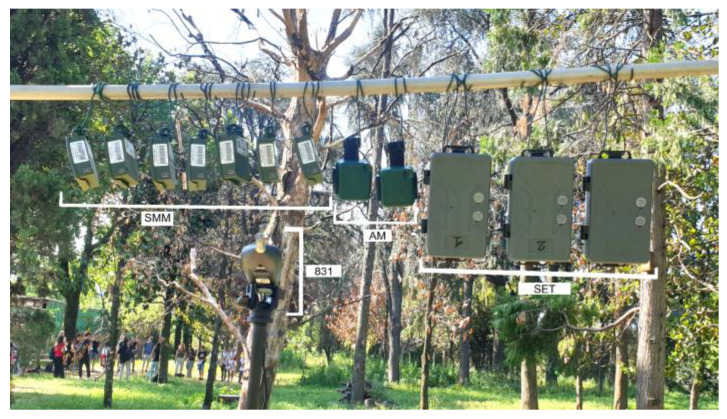
Devices placed in the urban park at a single measurement point.

This in-field validation was performed on a dedicated recording campaign carried out in June 2023 in a pocket park belonging to the University Campus, placing the devices at a single measurement point (Figure 11).

To obtain simultaneous recordings, each sensor was hung on a steel bar with the microphones oriented in the same direction. They were set with a sampling rate of 48 kHz (same gains used in the anechoic chamber, Table 1). The Audiomoths were used with their waterproof case.

Nine simultaneous 1 min recordings were acquired for each device. One of the Audiomoths did not work, and thus, it was not considered in the analysis.

Since the recorders were placed at a certain distance from the level meter, the recordings were examined to grant the registrations’ simultaneity. This operation was carried out in Audacity by selecting noticeable sound events and cutting the recordings with an accuracy at the millisecond level.

Validation was performed by calculating the eco-acoustic indices and Equations (1) and (2) and by comparing the indices calculated on the level meter with the ones of each soundscape sensor (using the three order values) through a statistical test. Given the simultaneity of the recordings, the Student pairwise test was carried out; the indices that did not meet the Student test assumptions (verified through Shapiro’s test and Bartlett’s test) were analyzed using the pairwise Wilcoxon test.

Just like for the 831′s white noise recording, the amplitude gain in the sound level meter was adjusted in the post-process phase to +18 dB.

##### Field Recordings in a Regional Park

**Figure 12:**
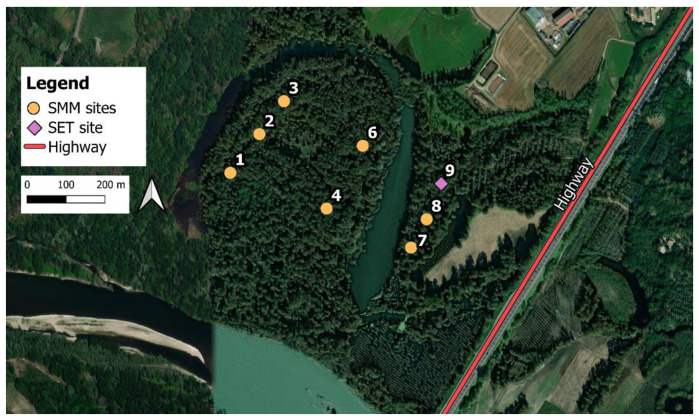
Monitoring scheme at the Ticino Regional Park (Bereguardo, PV, Italy). The SMMs are indicated as yellow circles while the SET as a violet triangle. Number 5 is missing due to malfunctioning. The main anthropic noise source (highway) is highlighted in red.

Using data acquired in the previous steps of this study, the best number of FFT points to use for each device was detected. These parameters were used for the equalization of a monitoring campaign (24 h) performed in April 2022 at the Ticino Regional Park, Lanca del Moriano (Bereguardo, Italy), using the same devices employed in the anechoic chamber (in particular, 7 SMM and 1 SET) placed as in Figure 12. The AMs were not employed since the monitoring campaign was organized only by our research group.

## 3. Results

### 3.1. Validation of White Noise Measures

**Figure 13:**
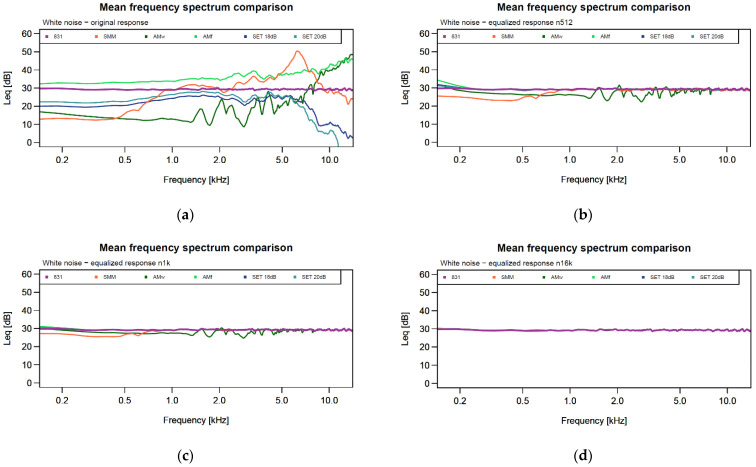
The white noise mean frequency spectrums of the four devices, before and after the equalization procedure. (**a**) The mean frequency spectrum of the original white noise recordings; (**b**) the mean frequency spectrum of the equalized white noise recordings using an order of 512; (**c**) the mean frequency spectrum of the equalized white noise recordings using an order of 1024; (**d**) the mean frequency spectrum of the equalized white noise recordings using an order of 16,384.

**Figure 14:**
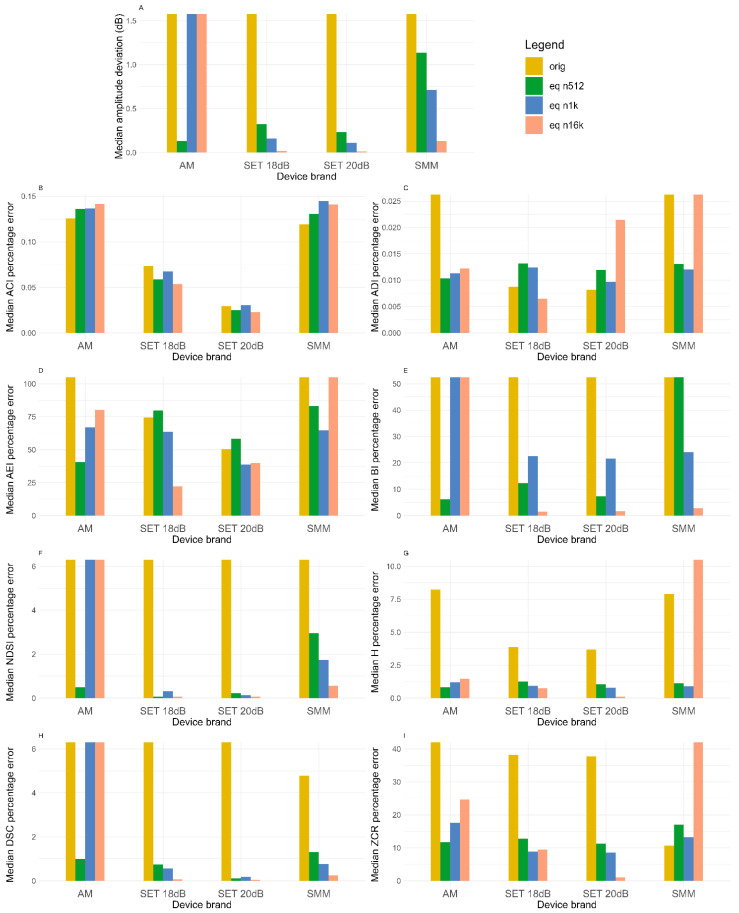
Barplots showing the effect of the equalization process on the amplitude RMSE and the percentage error of the eco-acoustic indices. Each graph represents a parameter ((**A**) median RSME, (**B**) median ACI percentage error, (**C**) median ADI percentage error, (**D**) median AEI percentage error, (**E**) median BI percentage error, (**F**) median NDSI percentage error, (**G**) median H percentage error, (**H**) median DSC percentage error, (**I**) median ZCR percentage error).The four devices (*x*-axis) and the three equalization options (colors) are reported.

In this section, the mean frequency spectrums of the four devices are compared, before and after the equalization procedure (Figure 13) distinguishing the three cases (512, 1024 and 16,384 order values). In addition to Figure 13, the amplitude RMSE and percentage error on eco-acoustic indices are shown in the form of barplots (Figure 14).

The mean frequency spectrums of the devices before the equalization (Figure 13a) show the evident difference between the level meter and the soundscape recorders. As observable in both Figure 2 and Figure 13, SMMs present a sensitivity peak at 6–7 kHz and a lower response at low frequencies than the level meter. On the other hand, the SETs are characterized by an almost flat response up to 6 kHz which then drops. Finally, the AMs without the waterproof case (AMf) present a nearly flat response up to 5 kHz, while the AMs equipped with the case (AMw) show an extremely oscillating response; differences in the AM frequency response depending on the case were also assessed by [46].

After the equalization (Figure 13b–d), the differences in mean frequency response between devices were reduced in terms of the overall dB difference (see *y*-axis) and sensitivity oscillation; the amplitude RMSE decreased from a mean value of 10.7 dB in the original recordings to 0.06 dB in the post-processed recordings using the 16,384-order filter (see Table 2). Thus, the best results are noticeable using a 1024- and 16,384-order filter for calculating the equalization curves (i.e., smaller fluctuations of the mean frequency spectrums and less overall amplitude deviation).

Below in Figure 14 are reported the amplitude RMSE and percentage error on the eco-acoustic indices distinguishing the devices and their status (original, post-process using 512, 1 k, 16 k order values). In the case of the AM, only the data about the waterproof-cased one are reported since it was used with this configuration in the field monitoring.

Examining Figure 14, the benefits produced are clear by looking at the reduction in the parameters from the original audios (yellow bar) to the equalized audios (green, blue and pink). The best improvements are seen in the AMs (with the case) and SMMs since they are the ones with the most variable frequency response (Figure 13). The amplitude RMSE (A) is reduced for each device. The percentage errors on the ADI, AEI, BI, NDSI, H and DSC are greatly reduced since they analyze the repartition of intensity on the spectrum (which depends on the frequency response). Table 2 shows that the total percentage error of the eight indices is reduced from over 1000% to almost 20%, with some indices (AEI, BI and ZCR) bearing the majority of the error (Figure 11).

Regarding the filter order, the AM equipped with the waterproof case presents less biases when a value of 512 is used, the SETs with the 16,384-order filter and the SMM with values of 512 or 1024 depending on the index (Figure 11, Table 2). These differences may be explained by the different sensitivity curves of devices in comparison with the level meter one.

### 3.2. Validation of In-Field Single Measurement Site (Urban Park)

**Figure 15:**
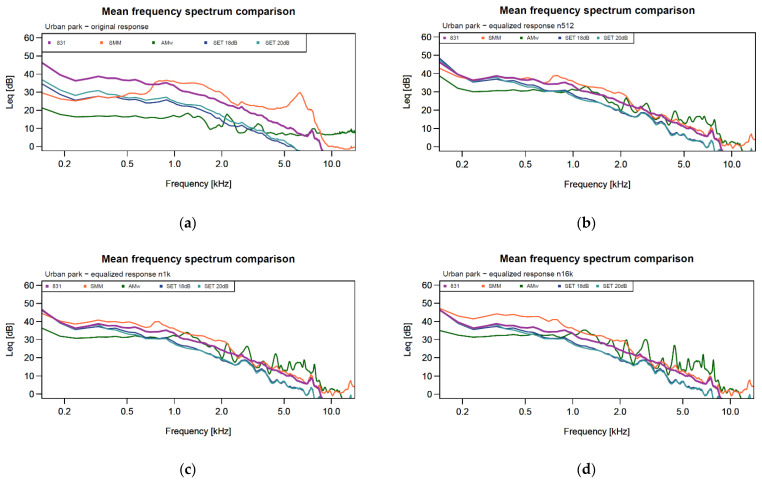
Urban park mean frequency spectrums of the four devices, before and after the equalization procedure. One of the nine recordings is displayed. (**a**) The mean frequency spectrum of the original in-field recording; (**b**) the mean frequency spectrum of the equalized in-field recording using an order of 512; (**c**) the mean frequency spectrum of the equalized in-field recording using an order of 1024; (**d**) the mean frequency spectrum of the equalized in-field recording using an order of 16,384.

This section mirrors the previous one, showing the mean frequency spectrums of the four devices, before and after the equalization procedure (Figure 15).

The original mean spectrums (Figure 15a) show the differences between devices. SMMs and the AM with the waterproof case (AMw) deviate more from the level meter (831) and respectively present a 6–7 kHz peak and an oscillating trend (like in the anechoic chamber). The SETs’ trends are more similar to the level meter. After the equalization (Figure 15b–d), the SMMs’ trends are very similar to the level meter, especially in the 512 and 1024 cases, while the SETs’ trends shift nearer to the reference curve. Finally, the AM is still affected by the oscillatory trend but is indeed nearer to the level meter curve. These benefits can also be seen in Table 3 where the amplitude RMSE decreases from a mean value of 11.6 dB in the original recordings to 4.5 dB in the post-processed recordings using the 512- and 1024-order filter and to 4.8 dB using the 16,384-order filter.

**Figure 16:**
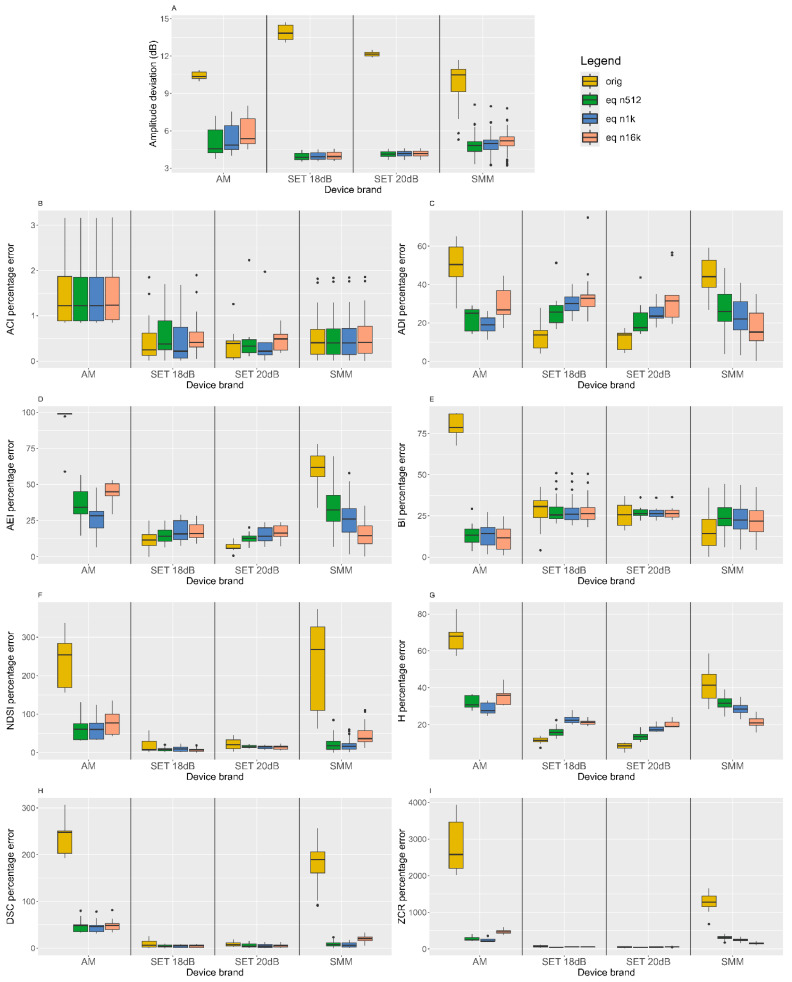
Boxplots of the amplitude RMSE (**A**) and the percentage errors on eco-acoustic indices (**B**–**I**). Each graph represents a parameter and the four measurement settings (original—equalized using orders of 512, 1024 and 16,384) are reported.

Mirroring the white noise validation section, Figure 16 shows the amplitude RMSE and percentage error on eco-acoustic indices calculated on the nine one-minute recordings carried out in the urban park.

The boxplot analysis on the amplitude RMSE and the percentage error on the eco-acoustic indices (Figure 16) reflects the barplot analysis of the white noise recordings (Figure 14). We can assess the following:

- The amplitude deviation is reduced for all devices (graph A);
- The percentage error on the BI, NDSI, DSC and ZCR is greatly diminished, in particular for the AM and SMM; in Table 3, it is possible to notice this decrease in the total percentage error: it is 2000% for AM and 1580% for SMM while only 30% for SET 18 dB and 2% for SET 20 dB;
- The ACI is not affected by the process since it compares adjacent temporal and frequency bins;
- The percentage error on the ADI, AEI and H is reduced in the SMM and AM, while it is increased in the SETs.

This comparison is summarized in Table 3; the total percentage error on the AM and SMM is reduced by 2000% and 1580%, respectively, and in this view, the optimal choice of parameters is using an order of 1024 for AM and 16,384 for SMM, while the error improvement on the SETs is limited from the outset and is not further reduced.

**Figure 17:**
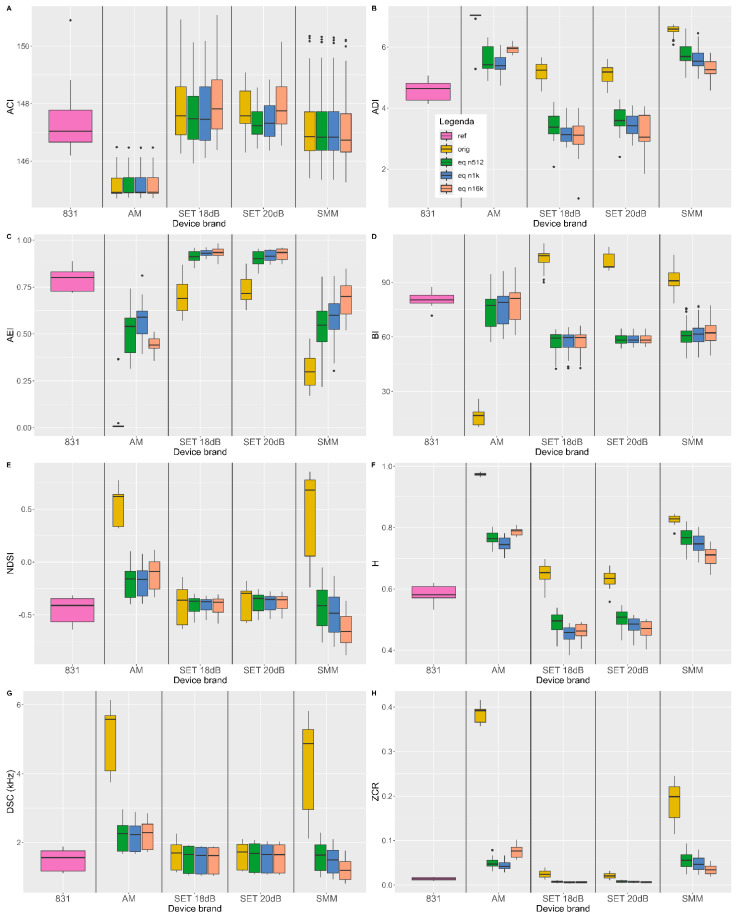
Boxplots showing the eco-acoustic indices. (**A**–**H**) Each graph represents an index and the four measurement settings (reference—original—equalized using orders of 512, 1024 and 16,384) are reported.

For a complete overview, Figure 17 shows the boxplot of the eco-acoustic indices calculated on the sound level meter and the soundscape recorders. The Student and Wilcox pairwise tests were performed on the eco-acoustic indices to better understand the benefits of the equalization procedure.

Examining Figure 17, we can affirm that the indices calculated on the in-field recordings behave as follows:

- ACI: The soundscape devices’ values are similar to the level meter ones due to the index calculation method. The AM is an exception probably due to its oscillatory frequency response. Looking at one device at a time, the variation in values between the original recordings and the equalized ones is constant or minor.
- ADI: The equalized values of AM and SMM are more similar to the level meter ones, especially in the 512–1024 case for AM and 16,384 for SMM. On the contrary, the SETs are similar to the reference in the beginning and then deviate. This different behavior between the soundscape devices may be explained by the linearity of their sensitivity curves and thus of the intensities on the spectrum: the “flatter” the curve, the smaller the deviation of ADI values from the level meter ones.
- AEI: As for the ADI, the AM and SMM values are nearer to the reference when equalized using a filter order of 1024 and 16,384, respectively. On the other hand, the SETs deviate when equalized. The deviation of the SET when equalized may be due to applying the procedure on a device which already presents a “flat” curve; trying to further linearize it generates errors since it is already linear.
- BI: All sensors’ original values do not match the reference ones; this can be explained by the frequency response of the soundscape devices that are not linear above 5 kHz. With the equalization, the AM values match the level meters’, and the others are nearer to the level meter ones.
- NDSI: Since this index greatly depends on the linearity of the frequency response, AM and SMMs are the ones with the greatest bias. It is possible to notice an improvement for all devices after the equalization, especially for AM and SMMs using an order value of 512.
- H: AM and SMMs benefit from the process but are nowhere near the reference values. The SETs’ original values are nearer to the level meter ones than the equalized values due to their already linear sensitivity curve. Moreover, the similar behavior is present in the ADI and AEI which also evaluate the heterogeneity of the recordings.
- DSC: AM and SMMs present the greatest biases due to their frequency response, as for the NDSI; the equalization reduces them, allowing for comparisons. The SETs do not benefit from the process thanks to their more linear frequency response.
- ZCR: Even in this case, AM and SMMs are affected by biases which are reduced with the equalization process. The SETs’ original values are very similar to the level meter ones probably due to their more linear sensitivity curve.

It is possible to observe a general trend in the SETs (i.e., ADI, AEI, H, DSC, ZCR) which is probably caused by their already almost “flat” sensitivity curve. When the SETs’ recordings are equalized, a deterioration in their similarity to the sound level meter is observed and caused by trying to further linearize their nearly linear frequency response.

To statistically validate the procedure, the eco-acoustic indices calculated on the level meter’s urban park recordings were confronted with the ones obtained with the soundscape recorders. In particular, each index series calculated on the nine one-minute-long level meter’s recordings were compared to the one calculated on each of the nine one-minute-long soundscape recordings before and after the equalization. For this reason, the tests used were the pairwise Student’s test (when both series were normally distributed and homoscedastic) or Wilcoxon’s test [45]. The tests’ null hypothesis H0 assumes that the mean difference in the distributions is equal to 0; it is refused when *p*-value < 0.05.

Looking at Table 4, the AM presents indices that confirm H0 (BI) when equalized. SETs present the same number of indices that confirm H0 when they are not equalized and equalized using the 512 and 1024 filter order. Finally, the SMMs benefit the most from the equalization when it is performed using an order of 1024 FFT.

### 3.3. In-Field Monitoring Campaign Example (Ticino Park)

**Figure 18:**
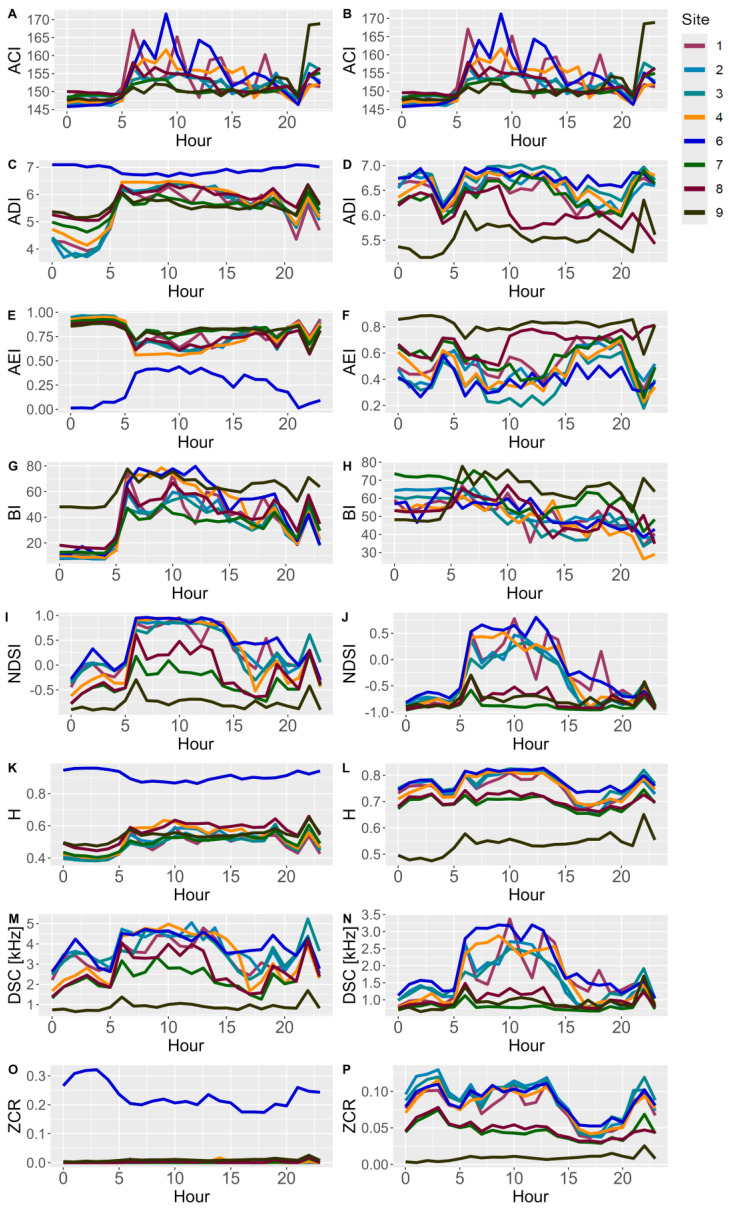
Time trend graphs of the eco-acoustic indices. Each row represents an index, on the left before the equalization and on the right after the equalization of the SMMs using a filter order of value 1024. Subfigures show: (**A**,**B**) time trend of ACI before-after the equalization, (**C**,**D**) time trend of ADI before-after the equalization, (**E**,**F**) time trend of AEI before-after the equalization, (**G**,**H**) time trend of BI before-after the equalization, (**I**,**J**) time trend of NDSI before-after the equalization, (**K**,**L**) time trend of H before-after the equalization, (**M**,**N**) time trend of DSC before-after the equalization, (**O**,**P**) time trend of ZCR before-after the equalization.

In this section, time trends of the eco-acoustic indices are shown (Figure 18). Given the results of the previous sections, the SMMs were equalized using a filter order of 1024, while the SET was not equalized (the AM was not used in this monitoring campaign).

In Figure 18, the indices’ time trends before (left column) and after (right column) the equalization are reported, and it is possible to affirm the following:

- ACI: the time trends remain the same since the index is not affected by the process.
- ADI: in the original time trends graph (C), the presence of the DC offset is noticeable which afflicts all the SMMs except Site 6 (blue curve); after the equalization, the SMMs present a similar trend.
- AEI: The DC offset is also visible in these graphs. The effect of the equalization is noticeable since the time trends are more similar in the post-equalization graph (F).
- BI: The SET (Site 9, black) is distant from the SMMs’ trends before the process (G) due to its flatter frequency response. After the equalization, the SMMs have a more similar trend to the SET.
- NDSI: The procedure reduces the SMMs’ overestimated biophonic contribution to the soundscape and the underestimation of the anthropophonies thanks to the equalization of the frequency response; in fact, values change from being almost constant at +1 for Sites 1–6 (SMMs) to a more oscillatory trend, while the anthrophonic disturbance generated by the highway becomes more evident in Sites 7–8–9 (SMM, SMM and SET).
- H: The DC offset is evident for this index just like the ADI and AEI (since it is not possible to define a low-frequency limit in these indices’ implementation in R). However, in the post-equalization graph (L), the SMMs’ trends are adjusted; given the results in the pocket park, these values may be overestimated.
- DSC: In the pre-equalization graph (M), the bias affecting the SMMs due to their peaked frequency response is extremely evident. After the equalization (N), time trends are corrected since Sites 7–8 (SMMs) are very similar to Site 9 (SET) indicating the influence of the highway (higher intensities at low frequencies); moreover, the general DSC values are reduced from a mean value of 4 kHz to 2.5 kHz, underlining the bias which afflicts this index if not corrected.
- ZCR: The DC offset is evident for this index. After the equalization (P), the values are corrected, and the difference between Sites 7–8–9 (nearer the highway) from the others is noticeable.

## 4. Discussion

The incongruence of eco-acoustic indices in analyzing the soundscape has been stressed by different studies, highlighting different trends [19,22] and limitations in predicting biodiversity parameters [21,47,48].

In particular, Alcocer and colleagues [47] showed the disparity in the number of studies published between 2007 and 2019 that found a correlation between eco-acoustic indices and biological parameters (i.e., avian richness) in favor of a lack of correlation between these two parameters. The absence of correlation was also confirmed by [21] through a meta-analysis involving four datasets from around the world; nevertheless, as the authors acknowledge in their paper, their findings may be explained with a non-harmonized data collection method in terms of the sampling design, bird survey method and recording devices [48].

Our study fits into this context, proposing an equalization protocol to mitigate the biases inherent in soundscape recordings and thus reducing the incongruence of eco-acoustic indices between studies. Nonetheless, the need for an accurate soundscape analysis is not recent, and other studies have covered this issue [24,25,26].

In [24], a calibration methodology is proposed, and, even if it is a different procedure than equalization, it highlights the need for more accurate soundscape analysis. Unfortunately, the sensitivity curves of the soundscape recorders are not linear; therefore, calibrating different devices will not obtain an equal output in terms of sound pressure levels and eco-acoustic indices’ values.

In [25], an equalization is performed on one-third octave bands between 25 Hz and 6.3 kHz using 1 s Leq values acquired with a type 1 sound level meter. This work lays a foundation for comparable recordings, but it is applied to a narrow frequency range (25–6300 Hz) using one-third octave bands, thus not covering the entire diversity of soundscape events (i.e., biophonies, anthropophonies and geophonies), and a single correction factor is used for a wide range of frequencies.

In our study, we performed an equalization procedure by taking as a reference the whole spectrum range using frequency ranges smaller than one-third octave bands thanks to the FFT analysis. Regarding this last point, and considering the validation stages conducted, we recommend using an order value of 1024 for equalizing the Song Meter Micro and 512 for the Audiomoth, while the Soundscape Explorer Terrestrial does not need to be corrected.

Finally, in [26], a pipeline to obtain similar eco-acoustic indices’ values between Audiomoth and Song Meter 4 (SM4) is proposed. The keystone of the process is the identification of comparable frequency ranges between the soundscape instruments (AM and SM4) on which the eco-acoustic indices are calculated. The approach led to a significant reduction of up to 70% in the variability observed in the calculation of eco-acoustic indices.

The selection of continuous comparable ranges suggested in [26] may not always be applicable since some devices may present oscillating sensitivity curves that do not allow for the selection of a continuous range and thus the implementation of eco-acoustic indices. Therefore, an equalization procedure is necessary to adjust the intensities throughout the entire spectrum allowing for the study of soundscapes in their integrity using virtually any recording device available on the market.

## 5. Conclusions

In this paper, we propose and validate an equalization procedure which we suggest implementing when studying soundscapes. In particular, it is extremely important when employing recorders characterized by a nonlinear sensitivity curve because it helps to reduce the biases that affect some eco-acoustic indices. Moreover, it reduces the errors when multiple brands of recorders are employed and when soundscapes from different habitats are compared.

Regarding the application of the procedure, we suggest applying it on devices that present a nonlinear frequency response. In our specific case study, we have proven that the equalization should be calibrated on device brands; moreover, each device should have its own equalization curve since there is a certain variance between devices of the same brand due to manufacturing processes. To calculate the equalization curve, the measurements of the reference signal should be carried out in a controlled environment, free-of-noise sources and with low reflectance (an anechoic chamber is the best choice). The MATLAB script of the procedure is available in the Supplementary Materials Section.

Future steps will involve its examination in different environments and the comparison between areas. Moreover, it will be applied to other soundscape sensors, and a new trial will be carried out using the add-on produced by Wildlife Acoustic to flatten the 6 kHz peak in the Song Meter Micro.

We hope this procedure will be adopted by the scientific community to enhance the understanding of the impacts of anthropic noise and the shaping of soundscapes, to help define guidelines and limits to effectively protect ecosystems and biodiversity.

## Figures and Tables

**Table 1:** Parameters set for each device in the anechoic chamber.

Parameters	831	SMM	SET 18 dB	SET 20 dB	AM
Sampling rate	48 kHz	48 kHz	48 kHz	48 kHz	48 kHz
Amplitude gain	+0 dB	+18 dB	+18 dB	+20 dB	+15 dB
Number of devices	1	7	2	1	2
Other characteristics	No windproof cap				With and without the waterproof case by Audiomoth

**Table 2:** The mean amplitude RMSE and total percentage error on the eco-acoustic indices calculated on white noise, specified for each device and status.

Device	Noise	Type	Mean Amplitude RMSE (dB)	Total Percentage Error ^1^
AM	White	Original	11.39	3777.26
		Eq n512	2.30	893.51
		Eq n1k	1.40	596.30
		Eq n16k	0.11	52.75
SET 18dB	White	Original	12.65	1567.79
		Eq n512	0.29	104.25
		Eq n1k	0.14	91.57
		Eq n16k	0.02	21.81
SET 20 dB	White	Original	10.27	1540.98
		Eq n512	0.23	78.12
		Eq n1k	0.11	70.15
		Eq n16k	0.01	42.86
SMM	White	Original	8.78	1445.55
		Eq n512	0.82	158.96
		Eq n1k	0.49	70.60
		Eq n16k	0.08	777.18

^1^ The total percentage error is calculated for each device at each status by summing the errors of the eight indices and averaging over the type of device.

**Table 3:** The mean amplitude RMSE and total percentage error on the eco-acoustic indices, specified for each device and status, in the urban park monitoring.

Device	Noise	Type	Mean Amplitude RMSE (dB)	Total Percentage Error ^1^
AM	Urban park	Original	10.42	3676.39
		Eq n512	4.99	500.41
		Eq n1k	5.30	440.31
		Eq n16k	5.81	729.19
SET 18 dB	Urban park	Original	13.89	165.49
		Eq n512	3.94	145.85
		Eq n1k	3.98	170.94
		Eq n16k	4.00	169.44
SET 20 dB	Urban park	Original	12.16	146.55
		Eq n512	4.12	144.50
		Eq n1k	4.14	158.51
		Eq n16k	4.15	173.74
SMM	Urban park	Original	9.95	1876.25
		Eq n512	4.81	454.86
		Eq n1k	4.90	374.68
		Eq n16k	5.07	288.19

^1^ The total percentage error is calculated for each device at each status by summing the errors of the eight indices and averaging over the type of device.

**Table 4:** *p*-values derived from Student’s and Wilcoxon’s tests performed between the eco-acoustic indices’ values of the level meter and the ones of each soundscape sensor. When H0 is confirmed (*p*-value > 0.05), the cell is highlighted in green. Here, we report the most interesting results from four devices.

Reference Device (Original)	Soundscape Device	Soundscape Device Status	ACI	ADI	AEI	BI	NDSI	DSC	H	ZCR
831	AM1	Original	3.91 × 10^−3^	3.91 × 10^−3^	3.91 × 10^−3^	1.93 × 10^−8^	1.74 × 10^−9^	3.91 × 10^−3^	3.91 × 10^−3^	3.91 × 10^−3^
		Eq n512	3.91 × 10^−3^	4.14 × 10^−6^	1.16 × 10^−5^	2.03 × 10^−1^	3.21 × 10^−5^	1.45 × 10^−5^	1.18 × 10^−10^	3.91 × 10^−3^
		Eq n1k	3.91 × 10^−3^	3.39 × 10^−6^	3.91 × 10^−3^	3.01 × 10^−1^	1.50 × 10^−5^	9.50 × 10^−6^	2.10 × 10^−10^	3.91 × 10^−3^
		Eq n16k	3.91 × 10^−3^	2.59 × 10^−6^	3.57 × 10^−7^	4.96 × 10^−1^	1.39 × 10^−6^	2.22 × 10^−6^	3.91 × 10^−3^	3.91 × 10^−3^
831	SET1 *	Original	8.41 × 10^−2^	3.51 × 10^−4^	1.97 × 10^−3^	2.28 × 10^−4^	4.32 × 10^−1^	1.95 × 10^−2^	2.72 × 10^−7^	3.91 × 10^−3^
		Eq n512	3.10 × 10^−1^	1.30 × 10^−5^	3.91 × 10^−3^	4.75 × 10^−5^	1.83 × 10^−2^	9.10 × 10^−1^	8.16 × 10^−9^	8.90 × 10^−7^
		Eq n1k	2.26 × 10^−1^	1.52 × 10^−7^	3.91 × 10^−3^	4.67 × 10^−5^	4.73 × 10^−2^	5.70 × 10^−1^	1.98 × 10^−11^	3.91 × 10^−3^
		Eq n16k	3.75 × 10^−2^	3.91 × 10^−3^	4.64 × 10^−6^	4.41 × 10^−5^	4.31 × 10^−2^	4.26 × 10^−1^	4.81 × 10^−13^	3.91 × 10^−3^
831	SET3 *	Original	6.47 × 10^−1^	7.38 × 10^−5^	6.26 × 10^−4^	2.27 × 10^−6^	1.65 × 10^−3^	3.76 × 10^−3^	1.03 × 10^−6^	3.91 × 10^−3^
		Eq n512	6.76 × 10^−1^	4.78 × 10^−5^	3.30 × 10^−6^	4.29 × 10^−7^	2.26 × 10^−5^	6.76 × 10^−2^	1.44 × 10^−8^	5.06 × 10^−7^
		Eq n1k	7.57 × 10^−1^	6.04 × 10^−7^	1.12 × 10^−5^	4.34 × 10^−7^	3.88 × 10^−4^	1.36 × 10^−1^	4.59 × 10^−11^	1.35 × 10^−6^
		Eq n16k	1.02 × 10^−1^	3.91 × 10^−3^	3.56 × 10^−6^	5.31 × 10^−7^	9.60 × 10^−4^	1.71 × 10^−1^	1.02 × 10^−11^	1.72 × 10^−6^
831	SMM1393	Original	1.07 × 10^−1^	3.91 × 10^−3^	3.99 × 10^−7^	9.44 × 10^−3^	1.95 × 10^−2^	3.91 × 10^−3^	3.91 × 10^−3^	3.91 × 10^−3^
		Eq n512	9.68 × 10^−2^	7.38 × 10^−5^	1.59 × 10^−4^	1.38 × 10^−5^	3.74 × 10^−1^	1.74 × 10^−1^	4.91 × 10^−11^	3.91 × 10^−3^
		Eq n1k	8.98 × 10^−2^	1.37 × 10^−4^	5.89 × 10^−4^	1.87 × 10^−5^	4.68 × 10^−3^	1.79 × 10^−1^	9.57 × 10^−11^	3.91 × 10^−3^
		Eq n16k	5.58 × 10^−2^	1.84 × 10^−3^	9.25 × 10^−3^	1.78 × 10^−5^	7.68 × 10^−5^	3.54 × 10^−5^	6.53 × 10^−10^	3.91 × 10^−3^
831	SMM1435	Original	5.65 × 10^−2^	1.55 × 10^−7^	9.64 × 10^−7^	3.91 × 10^−3^	3.91 × 10^−3^	3.91 × 10^−3^	3.91 × 10^−3^	3.91 × 10^−3^
		Eq n512	7.42 × 10^−2^	1.46 × 10^−5^	2.96 × 10^−5^	1.49 × 10^−5^	4.54 × 10^−2^	8.23 × 10^−3^	3.85 × 10^−11^	3.91 × 10^−3^
		Eq n1k	7.42 × 10^−2^	2.91 × 10^−5^	7.41 × 10^−5^	2.84 × 10^−5^	6.74 × 10^−1^	9.39 × 10^−1^	7.00 × 10^−11^	3.91 × 10^−3^
		Eq n16k	5.47 × 10^−2^	4.00 × 10^−4^	1.15 × 10^−3^	4.97 × 10^−5^	1.58 × 10^−9^	1.69 × 10^−7^	2.23 × 10^−9^	3.91 × 10^−3^

* SET 1 belongs to the SET 18 dB series; meanwhile, SET3 is the SET 20 dB.

## Data Availability

The raw data supporting the conclusions of this article will be made available by the authors on request.

## Supplementary Materials

The following supporting information can be downloaded at https://www.mdpi.com/article/10.3390/s24144642/s1.
