# Supplementary material for: Biases in Ecoacoustics Analysis: A Protocol to Equalize Audio Recorders
Source: Sensors (Basel). 2024 Jul 17;24(14):4642. doi: 10.3390/s24144642 (PMC11280489; doi:10.3390/s24144642)
Supplement: Supplementary file 1 [file sensors-24-04642-s001.zip › FINAL/equalization procedure script.pdf]

```
% Here it is reported the script to carry out the equalization procedure.
% The script is composed of two steps:
% 1.  Calculation of the equalization curve using white noise recordings
% 2.  Application of the equalization curve on in field recordings
% For the development of this script the MATLAB software version R2023a was used.



% STEP 1. Calculation of the equalization curve filter parameters (A and B) using white
noise recordings

    % A. import the white noise recordings

             % set the directory
             cd 'C:\Users\zzz\Documents\White noise recordings'

             % import white noise recorded with the reference device (SLM)
             [reference,Fs] = audioread('reference_whitenoise.wav'); % "reference" is
             %the name of the audio, "Fs" memorize the sampling frequency

             % import white noise recorded with the soundscape device
             [soundscape,Fs] = audioread('soundscape_whitenoise.wav'); % "soundscape"
             %is the name of the audio

             % calculate some parameters
             length_soundscape=length(soundscape);
             dt=1/Fs;
             t_soundscape = 0:dt:(length_soundscape-1)*dt; %creates a vector that
             %increments by "dt"
             t_reference = 0:dt:(length(reference)-1)*dt;
             fftLength=1024; % fft used to calculate Power Spectral Density (in the
             %article we used 512, 1024, 16384)
             filterorder=1024; % filter order used to calculate the equalisation curve
             %(in the article we used 512, 1024, 16384)


     % B. calculate the power spectral density (PSD) to use for the curve calculation

          % the formula is: [Pxx,f] = pwelch(x,window,noverlap,nfft,fs)
              % Uses the sampling frequency fs specified in hertz (Hz) to compute the
              % PSD vector (Pxx) and the corresponding vector of frequencies (f).
              % "x" indicates the audio (in this case the left channel)
              % "window" divides "x" into segments according to "window";
              % "ones(fftLength,1)" indicates that the audio is divided in "fftLength"
              %segments of value 1
              % noverlap=[] indica che si usa l'overlap di default (=50%) per calcolare
              %i segmenti di "x"
              % "nfft" indicates the fft value to be used
              % "Fs" indicates the sample rate value

             [PSDreference,Freq]=pwelch(reference,ones(fftLength,1),[],fftLength,Fs);

             [PSDsoundscape,Freq]=pwelch(soundscape,ones(fftLength,1),[],fftLength,Fs);


     % C. calculate the equalization curve (eq_curve=reference/soundscape)
             % it will be used into the fir2 function as the 'magnitude' to calculate
              % the filter curve parameters

             eq_curve=sqrt(PSDreference./PSDsoundscape);
```

```
% D. Calculate the filter curve parameters

  % the application of the curve to the in-field wav recording
  % is done using the 'filter' function (which uses a rational
  % transfer function defined by the coefficients B (numerator) and A
   % (denominator)), thus:

   % calculation of coefficients B and A

        % B:
        wn=Freq/max(Freq); % normalising the frequencies of the curve with respect
         % to the maximum value
        B=fir2(filterorder,wn,eq_curve); % B is the filter coefficient created with
         % the command 'fir2' which uses an order value (filterorder), on the
         % frequencies defined by 'wn' with magnitude 'eq_curve'.

        % A:
        A=1; % being A the denominator of the transfer function, setting it equal
             % to 1 does not distort the data


% E. save the parameters (B and A) in a .mat file for later use on field
     % recordings
   cd 'C:\Users\zzz\Documents\Eq filters'
   save Filter_deviceZZZ_1kfft_24hz15khz.mat B A




% STEP 2. Application of the equalization curve on in field recordings


% A. Supposing you have multiple in-field .WAV recordings, set the directory from
     % where the for cycle will load one file at a time and equalize it
     % NB. it works even if the folder contains only one audio file

   cd 'C:\Users\zzz\Documents\Recordings in field'

   % define file type (.wav)
   files = dir('*.wav');

   % get the number of wav files contained in the directory
   nfiles = length(files);

   % "double" item where the filenames that are non readable (and thus where not
    % equalized) are stored
   nonreadablefiles=[];


% B. Loading the filter curve .mat (B and A parameters)
   load('C:\Users\zzz\Documents\Eq filters\Filter_deviceZZZ_1kfft_24hz15khz.mat')

   filterorder=1024; % set the filterorder used to calculate B


% C. Run the for cycle
        % in this cycle, the DC offset correction is performed (C.1), the
        % equalization is carried out (C.2) and the time delay introduced by
        % the fir function is corrected (C.3). Moreover, a suffix to
```

```
        % the filenames is added to distinguish them from the original
        % recordings (C.4), save the new audio file (C.5) and a csv list is printed
         % with the non readable
        % original files that couldn't be imported by "audioread" (C.6)

    for i = 1:nfiles
       try % "try" is necessary for the C.5 step; when a file cannot be read "try"
           % skips the code
           % to the "catch" part and print the csv with the corruped filename
           % inside

        thisfile = files(i).name;           % read the i-th audio file in the
                                            % directory
        [wav_in,Fs] = audioread(thisfile);  % "wav_in" is the name of the audio,
                                            % "Fs" memorize the sampling frequency


        % C.1. DC offset correction (using the mean estimation method)
        wav_in_dc = wav_in - mean(wav_in);   % mean(wav_in) calculate the DC offset
         % entity which is then subtracted to the wav file; "wav_in_dc" is the audio
         % without the DC-offset


        % C.2. Equalizing the audio file
        wav_in_dc_EQ=filter(B,A,wav_in_dc);  % application of the equalization
         % curve filters using the "filter" function; "wav_in_dc_EQ" is the audio
          % equalized and without the DC-offset


        % C.3. Correction of the time delay introduced by the "filter" function
                % Filtering a signal introduces a time delay (i.e., the output
                % signal is shifted in time); this delay is calculated using the
                % "grpdelay" function and then corrected

        % calculation of the time delay
        x=length(wav_in);
        t_or = (0:length(wav_in)-1)/Fs;
        [gd,w] = grpdelay(B,A,filterorder);      % returns the n-point group delay
         % response vector gd and the angular frequency vector w for % the digital
         filter with transfer function coefficients stored in b and a.
        delay = mean(grpdelay(B,A,filterorder)); % get the mean delay value
        delay=round(delay);                      % to get corrected, the "delay"
                                                 % must be an integer (thus is
                                                 % rounded)

        % performing the time delay correction
        t_or_corr = t_or(1:end-delay);
        sn = wav_in(1:end-delay);
        wav_in_dc_EQ_corr = wav_in_dc_EQ;
        wav_in_dc_EQ_corr(1:delay) = [];


        % C.4. Adding the suffix "_EQ" to the filename
        filenameOR2=char(thisfile);                     % get the filename
        filenameS = strsplit(filenameOR2,'.');          % remove the .wav
        filenameS=filenameS(1);                         % get only the name
                                                        % (without ".wav")
        newfilename = fullfile([filenameS, 'EQ.wav']);  % suffix "EQ"
        filenameEQ = strjoin(newfilename,'_');          % merge the suffix to the
                                                        % filename and put a "_" in
                                                        % between
```

```
            % add the folder path to the filename to be saved
            filenameFinal=fullfile(['C:\Users\zzz\Documents\Recording in field
EQ\',filenameEQ]);


            % C.5. Save the corrected wav file (DC-offset corrected, equalizaed and
             % without delay)
            audiowrite(filenameFinal,wav_in_dc_EQ_corr,Fs); %set the new filename, what
             % audio to be saved


            % C.6. Print a csv list with the non readable original files that couldn't
                   % be read by "audioread"
                    % the csv is overwritten everytime a new non readable file is found
           catch exception

                % print this message with the name of the non readable file
                fprintf('Error in reading %s: %s\n', files(i).name, exception.message);

                % add the filename to the list
                nonreadablefiles=vertcat(nonreadablefiles,files(i).name);

                % print the csv file
                writematrix(nonreadablefiles, 'C:\Users\zzz\Documents\Recording in
field EQ\nonreadablefiles.csv', 'Delimiter', ',', 'QuoteStrings', false);

                continue;  % Move on to the next file in the directory without
                           % interrupting the cycle

           end

            disp(i);

        end
```
